# Addition to “Metallomimetic C–F Activation Catalysis by Simple Phosphines”

**DOI:** 10.1021/jacs.4c04291

**Published:** 2024-04-12

**Authors:** Sara Bonfante, Christian Lorber, Jason M. Lynam, Antoine Simonneau, John M. Slattery

We would like to bring readers’ attention to some recent work using tricyclohexylphosphine as a catalyst, where mechanistic pathways are proposed that share some common features with those reported in this paper.^1−3^ In particular, a fluorophosphorane intermediate is invoked, and the proposed catalytic cycles involve both P(III) and P(V) intermediates. Our oversight of these publications in our original Article was unintentional, and we hope that this addition will facilitate mechanistic comparisons for readers.
